# Global burden of cardiovascular diseases attributable to diet low in seafood omega-3 fatty acids from 1990~2021 and forecasting the future trends: A population-based study

**DOI:** 10.1371/journal.pone.0316767

**Published:** 2025-02-05

**Authors:** Qingsong Mao, Xinling Tian, Xingyi Wang, Haitao Xu, Yunyi Zhang, Yuzhe Kong

**Affiliations:** 1 Hepatobiliary Pancreatic Surgery, Banan Hospital Affiliated of Chongqing Medical University, Chongqing, China; 2 Xiangya School of Medicine, Central South University, Changsha, China; 3 College of Philosophy, Law & Political Science, Shanghai Normal University, Shanghai, China; National Center for Chronic and Non-Communicable Disease Prevention and Control, CHINA

## Abstract

**Background:**

This research investigates the worldwide influence of cardiovascular diseases (CVD) associated with low intake of seafood omega-3 fatty acids, based on data from the 2021 Global Burden of Disease Study.

**Method:**

The study evaluated the effects of insufficient seafood omega-3 fatty acid consumption on CVD at international, regional, and country levels. It analyzed variations across different demographics, including age and gender, and explored the relationship between disease burden and the Socio-Demographic Index (SDI). Additionally, it utilized an ARIMA model to predict the incidence of CVD connected to this dietary deficiency until 2050.

**Result:**

In 2021, diets deficient in seafood omega-3 fatty acids contributed to roughly 737.88 thousand deaths and 17.87 million disability-adjusted life years (DALYs) from CVD, with a noted decrease in this health impact over the period studied. The most substantial effects were seen in the elderly, particularly those aged 75 and above, with males experiencing a higher disease impact. Future forecasts suggest probable declines in disease rates across all SDI areas. However, nations in North Africa and the Middle East are projected to encounter growing difficulties related to CVD stemming from low seafood omega-3 intake by 2030 and 2050.

**Conclusion:**

These results highlight the critical need for preventive strategies against CVD and stress the significance of dietary management.

## 1. Introduction

Global data indicate that cardiovascular diseases (CVD) are leading causes of death, with the Mediterranean Diet’s seafood component crucial for heart health [1–3]. Studies show that eating fish regularly lowers risks of CVD and mortality [4–6]. Recent analyses suggest an additional 100 g/day of fish reduces heart issues, with moderate consumption improving overall health [5,7]. However, the impact on CVD mortality is minimal (RR: 0.96; 95% CI: 0.94–0.98) [8]. Notably, fatty fish consumption is linked to reduced CHD risk and death, attributed to high levels of beneficial fatty acids [3,9].

Dietary recommendations for CVD prevention differ, especially in terms of fish consumption. The American Heart Association suggests 1–2 seafood meals weekly to reduce CVD incidence and deaths [10]. The European Society of Cardiology recommends including fatty fish in at least one weekly meal [11]. However, concerns about pollutants in large fatty fish have led to mixed guidelines for consuming fish rich in n-3 fatty acids, especially among those at high CVD risk [9,12,13]. More long-term research is needed on the impact of high n-3 fatty acid consumption on CVD in healthy populations [14]. These findings are crucial for refining dietary guidelines focused on heart health [8].

This study utilized 2021 global disease burden data to assess the impact of low seafood omega-3 fatty acid diets on CVD and predicted future disease trends using the ARIMA model, validated by prior research [15].

## 2. Method

### 2.1. Study population

Our study utilized the 2021 Global Burden of Disease Study data to analyze 369 diseases and injuries and 87 risk factors across 204 nations from 1990 to 2021 [16].

To evaluate the burden of cardiovascular diseases (CVD), we used methodologies outlined in the literature [17]. We corrected data from various sources, including health surveys and verbal autopsies, to enhance accuracy [18]. The refined data fed into the Cause of Death Ensemble model (CODEm), producing annual, region-specific mortality rates for CVD by age and gender [17,19]. We performed a comparative risk assessment to identify significant CVD risk factors and calculated population attributable fractions (PAF) to measure the impact of low seafood omega-3 intake on CVD rates. These PAFs helped estimate CVD mortality and disability-adjusted life years (DALYs) for various demographics and time periods [15]. DALYs were calculated by adding years lost due to premature death (YLLs) from lower respiratory infections (LRIs) and years lived with disability (YLDs), adjusted by the severity of the condition [18]. We also developed the Socio-Demographic Index (SDI) using factors like total fertility under 25 (TFU25), educational attainment over 15 (EDU15+), and per capita income, classifying 204 areas into five SDI categories [18,20].

### 2.2. Statistical analysis

Age-adjusted rates (AAR) were used to standardize mortality and DALY metrics across countries with varying age distributions and demographics. We applied linear regression to the natural logarithms of these metrics, defined as y = α + βx + ε, where x represents the year, and y is the natural log of the rate. The estimated annual percentage change (EAPC) was derived using the formula 100 *  (e^β−1), with inclusion of a 95% confidence interval (95% CI). An upward trend in AAR was identified when the EAPC and the lower limit of the 95% CI were both positive, a downward trend when both the EAPC and the upper limit of the 95% CI were negative, and a stable trend was determined when neither condition was met [21,22]. We assessed the link between AAR and the Socio-Demographic Index (SDI) using Gaussian process regression with Loess smoothing, and performed Spearman rank correlation tests to analyze this relationship [20,22]. A decomposition analysis evaluated the impact of aging, population growth, and epidemiological shifts on DALY changes from 1990 to 2021, employing methods described in prior research [23].

Additionally, the ARIMA (Autoregressive Integrated Moving Average) model was employed to study the impact of a low seafood omega-3 fatty acid diet on CVD trends, and to forecast these trends globally, regionally, and nationally from 2020 to 2050. In the ARIMA model configuration (p, d, q), ‘AR’ signifies the autoregressive component with p denoting the number of lag observations, ‘MA’ indicates the moving average segment with q representing the lag of forecast errors, and d describes the differencing needed to stabilize the data [24]. Model selection was optimized using the Akaike Information Criterion (AIC) and the Bayesian Information Criterion (BIC).

Uncertainty intervals (UI) of 95% were calculated for all metrics. Rates were expressed per 100,000 population. All data management, analysis, and visualizations were executed in R software, version 4.3.2.

## 3. Result

### 3.1. Spatiotemporal patterns of CVD attributable to diet low in seafood omega-3 fatty acids

In 2021, diets deficient in seafood omega-3 fatty acids were linked to approximately 627.34 thousand deaths and 15.51 million disability-adjusted life years (DALYs) due to cardiovascular diseases (CVDs), with an age-standardized mortality rate (ASMR) of 7.4880 (95%UI, 1.4238–12.9487) and an age-standardized DALY rate (ASDR) of 181.0693 (95%UI, −36.1756–302.8448) per 100,000 population. The past thirty years have seen a significant reduction in the impact of CVD from diets low in omega-6 polyunsaturated fatty acids (Table 1, S1 Table).

**Table 1:** Global and regional deaths and DALYs of CVD attributable to diet low in seafood omega-3 fatty acids in 1990 and 2021 in 27 global regions.

Location	Deaths Number in 1990	Deaths Number in 2021	ASMR in 2021	DALY Number in 1990	DALY Number in 2021	ASDR in 2021
**Global**	500154.2790 (839562.7309, 98926.6596)	627342.2166 (1082744.9619, 119537.6607)	7.4880 (12.9487, 1.4238)	13048431.9384 (21367228.9035, 2692892.7487)	15511021.3514 (25946111.2991, 3098824.3958)	181.0693 (302.8448, 36.1756)
**Region**						
East Asia	58196.6574 (96907.7642, 12024.6169)	76918.7661 (140827.3363, 13850.5471)	4.1699 (7.7090, 0.7683)	1635332.6369 (2711932.2037, 348971.6615)	1545028.7134 (2818403.9246, 287279.5919)	77.7272 (139.9985, 14.4650)
Southeast Asia	18769.2285 (32147.9024, 3604.1314)	15038.9802 (27865.2892, 2883.0509)	2.5172 (4.7425, 0.4653)	575959.7219 (969726.8579, 117766.3663)	422055.2867 (766793.4499, 86398.9278)	61.3666 (112.7415, 12.3267)
Oceania	261.7617 (464.5550, 52.9117)	589.0915 (1064.5450, 107.6229)	7.8139 (14.2763, 1.3306)	8883.2639 (15577.0650, 1819.6820)	19845.7082 (35969.2998, 3822.6981)	211.9113 (380.4434, 38.9190)
Central Asia	18310.2562 (30364.5935, 3785.9876)	23071.6264 (39584.4159, 4471.6119)	32.7876 (56.6775, 6.2330)	447303.4249 (726967.7716, 96199.5730)	550603.5537 (916554.8746, 111185.9045)	678.3957 (1146.5798, 134.2078)
Central Europe	39235.5282 (67036.8807, 7594.7492)	26956.1801 (47814.3348, 4937.6790)	11.6812 (20.6125, 2.1526)	899556.7541 (1494646.4724, 182507.9836)	487348.3088 (846804.9756, 90553.4363)	228.7111 (393.8513, 43.0327)
Eastern Europe	51894.0258 (91828.9416, 9323.2328)	48935.3874 (90738.4178, 9527.1032)	13.8586 (25.6304, 2.7123)	1113120.9835 (1938815.7265, 212359.3683)	924250.8400 (1696746.3688, 183502.9798)	272.1946 (495.8115, 54.1763)
High-income Asia Pacific	551.8062 (1184.4290, 101.0511)	150.3835 (365.2131, 21.9135)	0.0247 (0.0585, 0.0039)	12129.0284 (24940.2278, 2255.7939)	2225.5716 (5269.5379, 367.2826)	0.5153 (1.1648, 0.0993)
Australasia	2501.2316 (4440.4131, 436.2828)	1106.7624 (2120.1372, 199.7193)	1.8298 (3.4294, 0.3415)	50274.8235 (86442.9324, 9070.7562)	17871.2645 (33299.6163, 3382.5774)	33.9897 (62.0440, 6.5511)
Western Europe	51278.8832 (92194.7849, 9330.7543)	22256.5207 (41153.5313, 4120.8057)	1.9682 (3.5836, 0.3696)	975632.8561 (1728897.3716, 184136.7591)	346695.4643 (623582.5387, 66517.4202)	37.0164 (65.7079, 7.2186)
Southern Latin America	6633.0515 (11458.5370, 1293.5136)	4056.5199 (7057.1497, 757.4984)	4.5592 (7.9162, 0.8542)	149908.0094 (255041.3356, 29977.8677)	84872.6929 (148119.3641, 16148.6904)	99.6697 (173.6875, 19.0532)
High-income North America	43950.4915 (78841.3321, 8071.9487)	38288.6191 (69406.7325, 7167.3235)	5.6737 (10.1738, 1.0904)	857455.9959 (1491369.1746, 160621.1251)	760480.9846 (1325751.0967, 148752.5966)	125.9406 (215.9776, 25.0105)
Caribbean	4088.5889 (6975.5960, 778.2633)	5204.4511 (9208.6342, 1047.5157)	9.6070 (16.9966, 1.9382)	101989.5602 (170194.3660, 20376.4386)	129392.4937 (222421.4781, 27318.2235)	242.3667 (416.2596, 51.2938)
Andean Latin America	1633.8124 (2810.7938, 323.2224)	2475.4047 (4428.8723, 458.4885)	4.2936 (7.7348, 0.7897)	42620.7664 (72160.2437, 8993.4948)	58014.3797 (100510.3002, 11263.8686)	95.7169 (166.2002, 18.3919)
Central Latin America	9488.1105 (16098.0096, 1876.7540)	21455.6970 (37692.7319, 4130.8201)	8.8589 (15.6210, 1.6930)	246492.7218 (406879.6408, 50523.5231)	493753.8101 (845868.2431, 100380.5236)	194.7427 (333.6033, 39.3600)
Tropical Latin America	12645.2723 (21306.4959, 2502.8052)	14918.1113 (25917.7316, 2882.2292)	5.8535 (10.1714, 1.1316)	359990.1133 (592881.9796, 75459.9518)	395826.5471 (664081.7501, 77855.5965)	151.3853 (254.6183, 29.7577)
North Africa and Middle East	51441.7635 (84668.8811, 10882.2377)	76675.3810 (131538.4087, 15165.4434)	18.7553 (32.6927, 3.6389)	1474542.5355 (2400300.8324, 322483.4338)	2061239.0025 (3462638.8529, 431035.7987)	420.8404 (714.2206, 85.0970)
South Asia	110984.5599 (181623.2301, 24467.7796)	214318.3589 (359006.9466, 42397.7897)	15.0919 (25.5382, 2.9352)	3580359.9646 (5736505.8149, 821322.7559)	6226485.9318 (10237060.5280, 1284359.6642)	391.0697 (647.8572, 79.5419)
Central Sub-Saharan Africa	2172.3172 (4029.8491, 427.4356)	4927.9041 (8922.1242, 895.2529)	10.5657 (19.3399, 1.9001)	62444.9840 (116293.6761, 12918.2238)	141970.7698 (257036.3005, 26092.6164)	238.8878 (432.9798, 43.4432)
Eastern Sub-Saharan Africa	6121.6867 (10394.8502, 1317.7838)	11922.2218 (19747.5078, 2462.0970)	7.6484 (12.8639, 1.5320)	187594.7822 (312497.5443, 42415.3699)	358762.7407 (590343.9340, 77399.6702)	184.7274 (306.2391, 38.5048)
Southern Sub-Saharan Africa	2251.7045 (3843.5134, 485.9489)	4761.5116 (8117.6650, 974.1532)	9.1672 (15.9249, 1.8221)	66273.9314 (110110.4849, 14972.5256)	131674.1691 (218082.4882, 28275.6539)	214.7054 (360.6239, 45.0403)
Western Sub-Saharan Africa	7743.5413 (13545.9492, 1559.9461)	13314.3380 (23059.0786, 2771.5438)	8.0388 (14.0706, 1.5893)	200565.0805 (348000.7942, 41838.3961)	352623.1182 (600724.0650, 77883.7469)	171.1389 (295.3907, 36.0188)
**SDI**						
High-middle SDI	128034.1787 (219711.8908, 24155.6535)	118535.4476 (213000.1835, 22449.5569)	6.1979 (11.1192, 1.1783)	3000474.1371 (5032740.0953, 589834.5008)	2304567.1787 (4001666.3862, 460293.3918)	120.5908 (208.4636, 24.3152)
High SDI	109595.6124 (196382.8553, 20233.7545)	70159.6821 (127778.9225, 12960.0905)	3.1399 (5.6254, 0.5926)	2202458.8891 (3856414.1725, 417123.4984)	1357895.8357 (2387431.4795, 262561.2073)	71.5689 (123.1859, 14.2564)
Low-middle SDI	104022.8922 (170364.7118, 21965.3390)	178266.4591 (297386.5761, 34896.2289)	13.0057 (21.9205, 2.5118)	3218936.1412 (5148423.1215, 713724.7977)	5101652.6918 (8327592.6938, 1029857.8907)	329.2352 (541.4672, 65.5120)
Low SDI	34358.2953 (57641.1644, 7304.3727)	65211.1099 (109236.2377, 13453.2539)	13.9332 (23.8449, 2.7656)	1040362.1728 (1727704.4836, 230710.8998)	1909743.3934 (3115474.5159, 409528.2534)	336.6821 (559.6893, 70.3037)
Middle SDI	123257.3162 (203006.8755, 25239.0307)	194451.1417 (338121.6184, 36791.4123)	7.9460 (13.9414, 1.4863)	3565185.6597 (5752071.4714, 755829.7469)	4821649.4129 (8042547.5548, 964067.3139)	179.7904 (302.4057, 35.4253)

**Fig 1:**
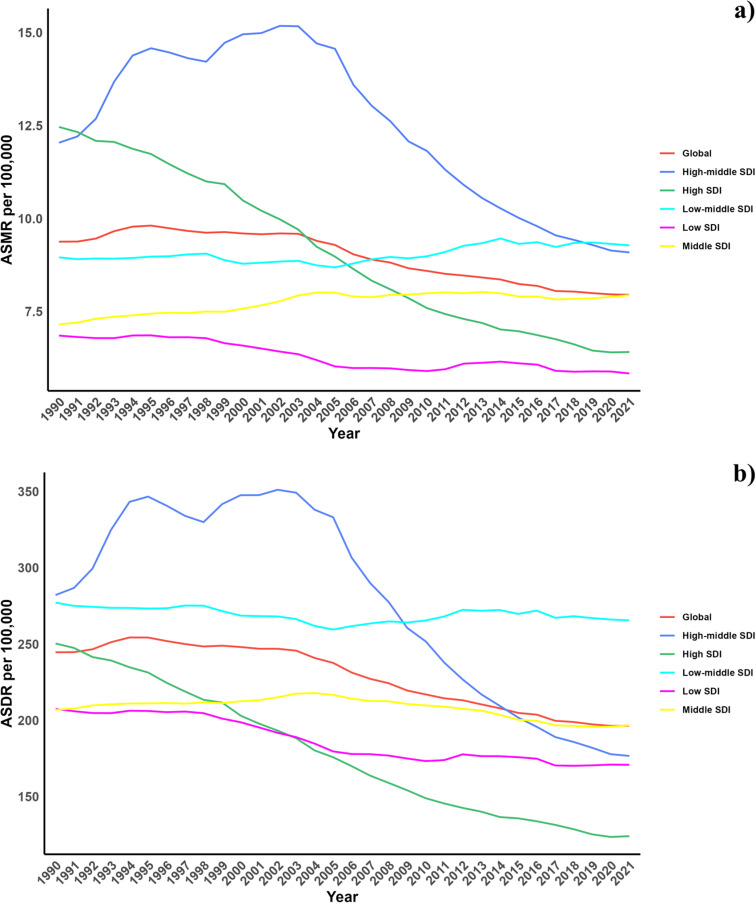
Temporal trends of ASMR and ASDR of CVD attributable to diet low in seafood omega-3 fatty acids from1990 to 2021 in different SDI regions.

Regarding the Socio-demographic Index (SDI), areas with higher SDIs have seen considerable declines in the CVD burden from diets low in seafood omega-3 fatty acids, with the most substantial reductions recorded. All SDI regions experienced modest reductions in both ASMR and ASDR for CVDs linked to diets low in omega-6 polyunsaturated fatty acids, except in the high SDI regions. However, the low-middle and middle SDI regions reported a slight increase in ASMR (Table 1, S1 Table, Fig 1).

Regionally, the most substantial CVD burdens from diets low in seafood omega-3 fatty acids were observed in Asia, high-income North America, North Africa, the Middle East, and Europe, with North Africa and the Middle East showing the highest values of ASMD and ASDR. Conversely, high-income Asia Pacific recorded the lowest CVD burdens associated with these diets (Table 1).

**Fig 2:**
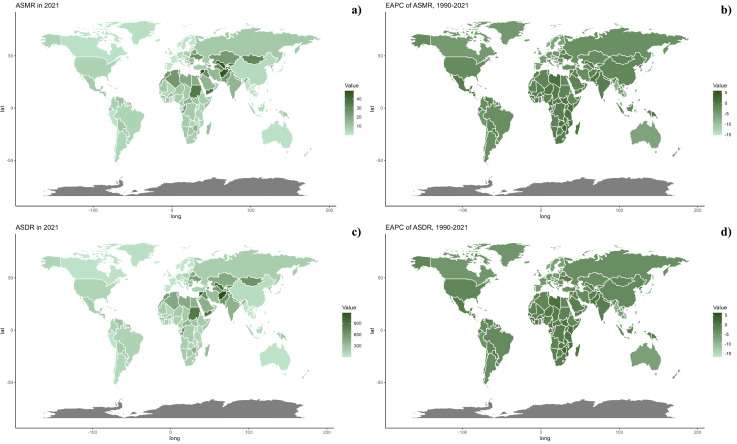
Global distribution of ASMR (a) and ASDR (c) of CVD attributable to diet low in seafood omega-3 fatty acids for both sexes in 2021 in 204 countries and territories. EAPC of ASMR (b) and ASDR (d) of CVD attributable to diet low in seafood omega-3 fatty acids from 1990 to 2021 in 204 countries and territories.

**Fig 3:**
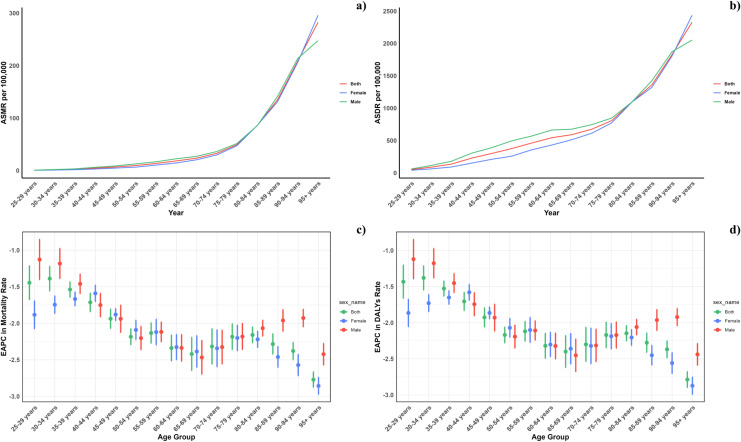
Age-specific rates of global deaths (a) and DALYs (b) of CVD attributable to diet low in seafood omega-3 fatty acids, by sex, in 2021 and the corresponding EAPC of global deaths (c) and DALYs (d) from 1990 to 2021.

At the national level, in 2021, the ASDR and ASMR for CVDs linked to diets low in seafood omega-3 fatty acids varied significantly worldwide, with the highest levels seen in the North Africa and Middle East regions (Fig 3). From 1990 to 2019, a general decline in both ASMR and ASDR was observed in most countries, with the exception of those in the North Africa and Middle East region (Table 1, S1 Table, Fig 2).

### 3.2. Age and gender pattern

Fig 3 displays the global age-specific mortality and DALY rates for CVDs in 2021, along with their trends from 1990 to 2021. The chart shows a J-shaped curve, indicating rising mortality and DALY rates in individuals younger than 75 years, with a notable increase observed in those aged 75 to 95 and older. In every age group, mortality rates from diets low in seafood omega-3 fatty acids were consistently higher in males compared to females, except for the group over 95 years where men exhibited lower mortality rates. Similarly, DALY rates related to such diets followed the same pattern. Moreover, the reduction in mortality and DALY rates from 1990 to 2021 was more modest among males, especially in those under 40 or over 75 years old.

**Fig 4:**
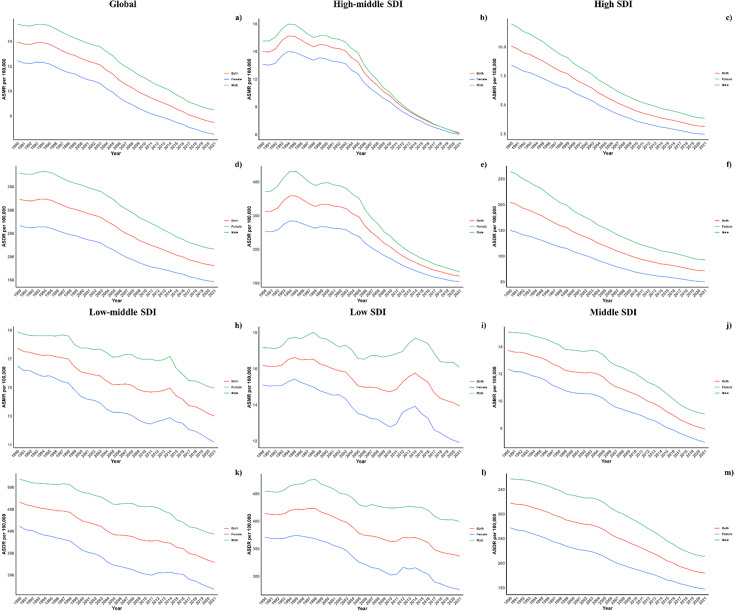
Sex Disparity in CVD attributable to diet low in seafood omega-3 fatty acids across SDI regions.

Across all Socio-demographic Index (SDI) categories, male mortality and DALY rates were consistently higher, maintaining this gender disparity across the regions. However, in regions with high-middle and high SDI, the differences between genders have lessened, whereas in other SDI regions, the disparities have either remained unchanged or increased (Fig 4).

### 3.3. Association with the socio-demographic index

**Fig 5:**
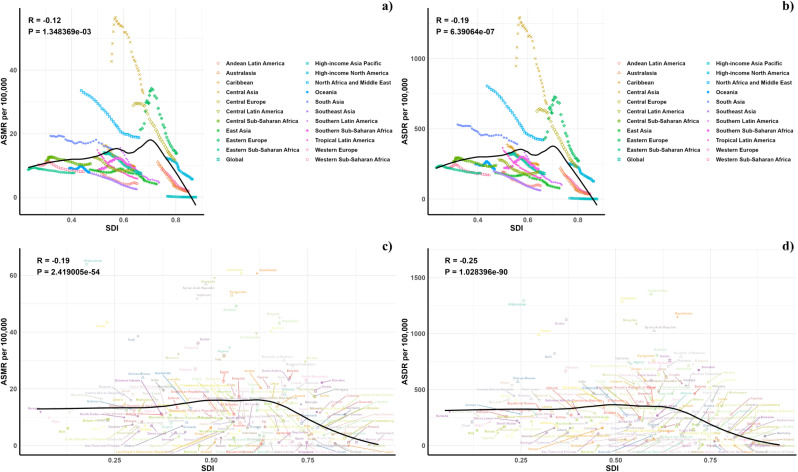
Correlations between ASMR (a, c) and ASDR (b, d) of CVD attributable to diet low in seafood omega-3 fatty acids and SDI at the regional level (a, b) and the national level (c, d).

Fig 5 illustrates a comparative analysis of both observed and predicted age-standardized DALY (ASDR) and mortality rates (ASMR) for CVD associated with diets low in seafood omega-3 fatty acids, mapped against Socio-Demographic Index (SDI) values at regional and national scales from 1990 to 2021. There was a discernible negative correlation between ASDR and rising SDI, suggesting a decrease in disease burden as SDI increases. Regions such as Eastern Europe, Central Asia, North Africa, and the Middle East exhibited higher-than-expected ASDR within this period. Similarly, the trend in observed versus forecasted ASMR based on SDI at regional levels mirrored the ASDR findings. Fig 5 also showcases the observed versus anticipated ASDR and ASMR at the national level for 2021, revealing a similar negative correlation with SDI values, both regionally and nationally.

### 3.4. Forecasts for the mortality, DALYs rate, ASMR and ASDR of CVD attributable to diet low in seafood omega-3 fatty acids in global (2022–2050)

**Fig 6:**
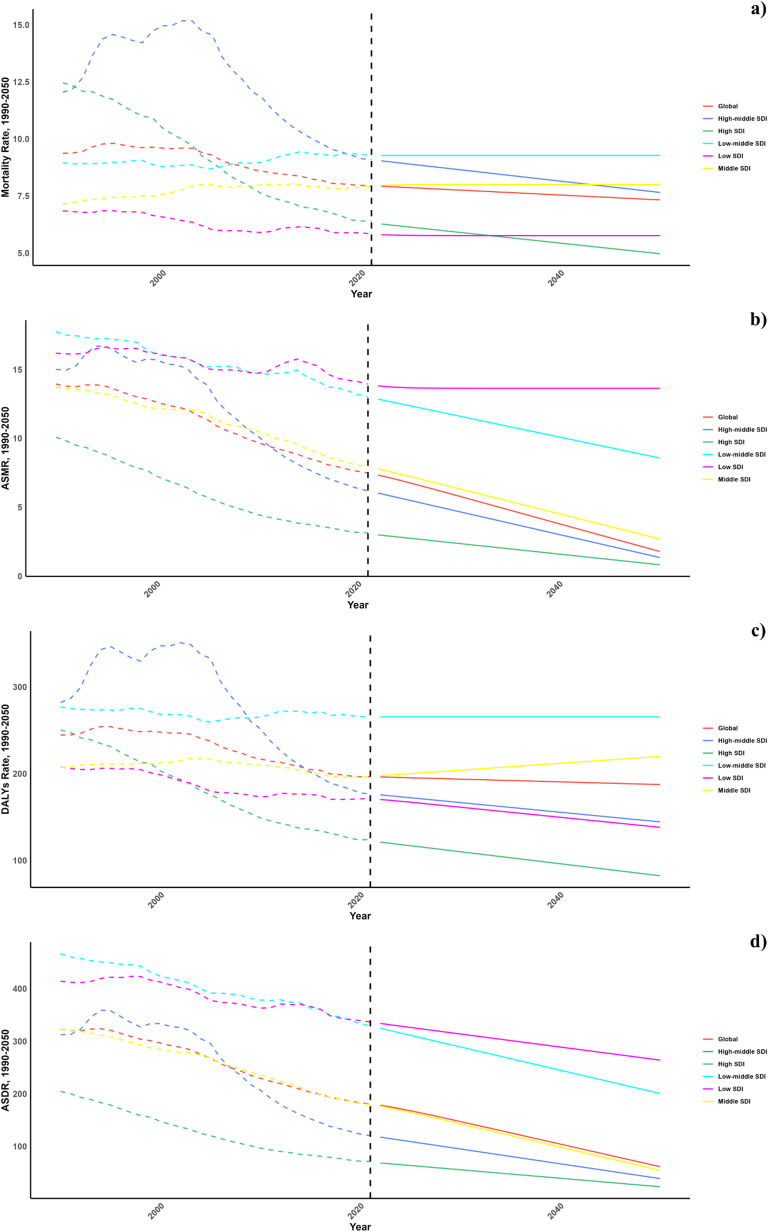
Estimated trends of mortality rate (a), DALYs Rate (b), ASMR (c) and ASDR (d), 1990–2050 at the regional level.

**Fig 7:**
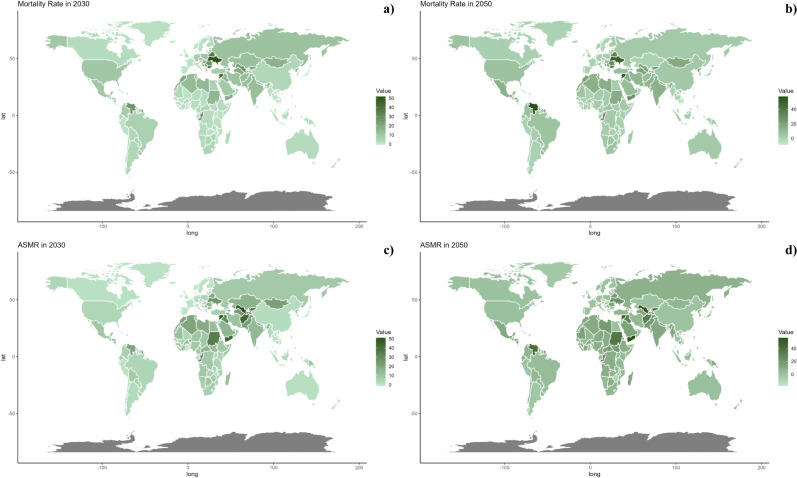
Estimated trends of mortality rate (a, b) and ASMR (c, d) in 2030 (a, c) and 2050 (b, d) at the national level.

**Fig 8:**
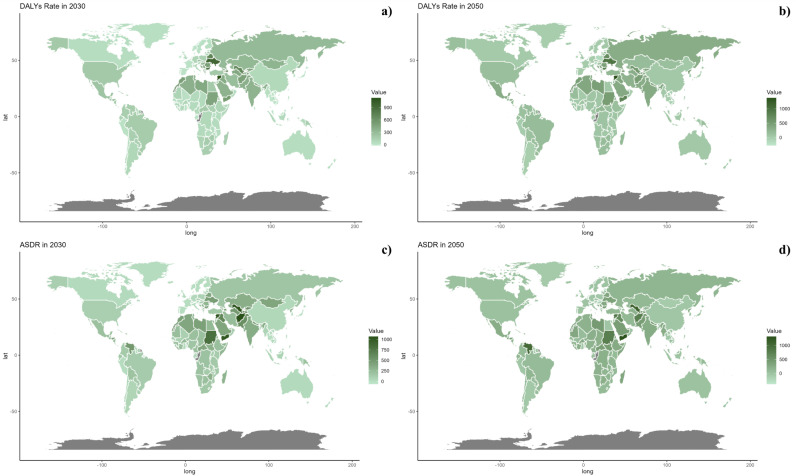
Estimated trends of DALYs Rate (a, b) and ASDR (c, d) in 2030 (a, c) and 2050 (b, d) at the national level.

Projections for mortality and DALY rates, including age-standardized mortality rate (ASMR) and age-standardized DALY rate (ASDR), related to cardiovascular diseases (CVDs) stemming from a diet low in seafood omega-3 fatty acids are presented in Figs 6–8. Regionally, a decline in CVD burden is expected across all Socio-Demographic Index (SDI) regions, except for the low SDI region, which is forecasted to maintain current mortality and ASMR levels.

On a national scale, the projections indicate that the patterns will remain fairly consistent through the years 2030 and 2050. Nevertheless, the anticipated impact of CVDs linked to insufficient intake of seafood omega-3 fatty acids is projected to be significantly greater in countries across the North Africa and Middle East region compared to other regions for both 2030 and 2050.

## 4. Discussion

This study examined the worldwide effects of cardiovascular disease (CVD) associated with a diet low in seafood omega-3 fatty acids from 1990 to 2021, identifying a significant decline throughout this period. The demographic most impacted included individuals aged 75 and older, with males showing higher incidence rates. Future projections indicate possible reductions in mortality across all SDI regions, yet a persistently higher burden of CVD related to diets low in seafood omega-3 fatty acids is expected in countries within the North Africa and Middle East region. This analysis represents the first extensive review of the impacts of such dietary deficiencies on CVD.

In Europe, fish and seafood consumption is diverse; white fish constitutes 49% and 45% of the total intake among women and men, respectively. Mediterranean countries like Greece show the highest diversity and rates of fish consumption, yet Northern Europe and Scandinavia report the most significant intake of fatty fish [25]. In Greece, adherence to national dietary guidelines is low, with only 30% and 10% of people meeting seafood and small fatty fish recommendations, respectively [26].

Dietary guidelines for CVD prevention typically recommend 1–2 seafood meals weekly. The American Heart Association (AHA) also endorses omega-3 PUFA supplements for individuals at risk of CVD, including those with coronary heart disease, type 2 diabetes, and heart failure [10,14]. Yet, the effectiveness of consuming n-3 fatty acid-rich fish versus total seafood for preventing CVD remains under debate, especially with concerns over contaminants in large fatty fish [9,13,27]. Mixed results from clinical trials on omega-3 supplementation in healthy individuals necessitate further research to clarify the potential benefits of high n-3 fatty acid fish intake in CVD prevention [8,28].

Comprehensive reviews have shown that increased fish consumption lowers the incidence of various CVD events by 12%–25%, including heart failure (SRR: 0.80; 95% CI: 0.67–0.95), myocardial infarction (SRR: 0.75; 95% CI: 0.65–0.93), and coronary heart disease (CHD) (SRR: 0.88; 95% CI: 0.79–0.99) [6]. However, high intake of n-3 rich fish showed no significant 10-year CVD reduction in the general population, though it did in high-risk groups [9,29].

For CVD mortality, significant declines were observed in individuals consuming more than two servings of marine fish weekly, especially among higher-risk groups like women and the elderly. Surprisingly, high consumption of small n-3 fatty acid-rich fish even reduced CVD mortality in healthy, normotensive individuals. Earlier studies indicated a modest 4% reduction in CVD mortality risk for healthy people (RR: 0.96; 95% CI: 0.94–0.98) [8], with an additional 100 g/day of fish linked to a 25% mortality reduction [6]. These findings highlight specific impacts of n-3 fatty acid-rich fish on 10-year CVD mortality previously undocumented.

As individuals age, typically over 65, lifestyle shifts occur, including reduced physical activity due to mobility issues and dietary changes often influenced by swallowing difficulties or financial constraints, leading to a diminished intake of varied foods, including seafood [30–33]. Increasing seafood and small fish consumption could notably benefit seniors.

The health benefits of consuming small fish rich in n-3 fatty acids, such as EPA and DHA, are significant, enhancing cardiometabolic health through their roles in lowering triglycerides, improving vascular function, offering antithrombotic effects, and reducing oxidative stress and inflammation [34–36]. These fatty acids help regulate lipoprotein levels, blood coagulation, and blood pressure, which are essential for maintaining cardiovascular health [36,37]. Prior research shows that regular fish consumption can lower levels of inflammatory markers like IL-6, CRP, and TNF-alpha [37], supporting reduced inflammatory responses and potentially preventing CVD progression. A recommended intake of around 1.0 g/day of n-3 fatty acids has been linked to the lowest cytokine production, with one serving per week of small fatty fish significantly benefiting CVD outcomes [3,38].

Evidence from studies on fish consumption, clinical trials on fish oil supplements, and related research suggests that regular fish intake might lower the risk of coronary heart disease, although its effect on overall CVD events is less clear [39,40]. Analysis from international cohorts shows that even minimal fish consumption (around 175 g weekly) correlates with reduced CVD events and mortality in those with previous CVD, but not generally [29]. Trials like the VITAL study on n-3 supplementation found no significant benefits in preventing major CVD events over 5.3 years in the general population without prior low fish intake, highlighting the need for public health strategies promoting n-3 rich seafood consumption across all demographics [35].

This extensive study faces several limitations that might affect the thoroughness and accuracy of its conclusions. A major constraint is the variable quality and availability of regional data, especially from less developed areas like Sub-Saharan Africa. The use of mathematical models to estimate cardiovascular disease burdens in these regions introduces significant uncertainty. Moreover, the study’s timeframe from 1990 to 2021, while providing a long-term view, may not fully reflect recent dietary shifts or the impact of latest public health interventions on the global CVD burden. This temporal gap suggests that recent changes in dietary habits and health policies may not be accurately represented in the study’s outcomes.

## 5. Conclusion

This research provides a comprehensive evaluation of the global impact of CVD related to diet low in seafood omega-3 fatty acids from 1990 to 2021, noting a notable decline during this period. The most affected demographic group was individuals over 75, particularly men who bore a heavier burden. Future projections indicate that all SDI regions may witness reductions in mortality rates, DALY rates, ASMR, and ASDR. On a country level, countries throughout the North Africa and Middle East region to maintain a high CVD burden associated with diet low in seafood omega-3 fatty acids through 2030 and 2050.

These findings are critical for formulating prevention strategies for CVD and emphasize the importance of managing dietary habits.

## Supporting information

S1 Table Global and regional deaths and DALYs of CVD attributable to diet low in seafood omega-3 fatty acids in 1990 and 2021 in 204 nations. (DOCX)
