# Supplementary material for: Global burden of cardiovascular diseases attributable to diet low in seafood omega-3 fatty acids from 1990~2021 and forecasting the future trends: A population-based study
Source: PLoS One. 2025 Feb 5;20(2):e0316767. doi: 10.1371/journal.pone.0316767 (PMC12051498; doi:10.1371/journal.pone.0316767)
Supplement: S1 Table — (DOCX) [file pone.0316767.s001.docx]

Table S1 Global and regional deaths and DALYs of CVD Attributable to Diet Low in Seafood Omega-3 Fatty Acids in 1990 and 2021 in 204 nations

| Location | Deaths Number in 1990 | Deaths Number in 2021 | ASMR in 2021 | DALY Number in 1990 | DALY Number in 2021 | ASDR in 2021 |
| --- | --- | --- | --- | --- | --- | --- |
| China | 56638.0466 (94407.2641, 11739.1710) | 72410.2424 (133501.9420, 13118.6296) | 4.1187 (7.6337, 0.7643) | 1592558.2524 (2656747.4525, 340659.8250) | 1428781.4914 (2626045.2234, 265229.0893) | 74.9000 (135.8970, 13.9067) |
| Democratic People's Republic of Korea | 1323.5617 (2391.2060, 260.9093) | 4383.3159 (7636.1596, 818.2184) | 14.5617 (25.1614, 2.6486) | 37292.5979 (65729.0635, 7944.0962) | 113890.5564 (198968.5741, 23030.8569) | 349.3017 (610.0287, 69.5469) |
| Taiwan (Province of China) | 235.0491 (454.8545, 39.1223) | 125.2078 (272.1274, 21.5918) | 0.2877 (0.6214, 0.0502) | 5481.7866 (10421.7160, 955.6796) | 2356.6655 (4976.8315, 440.4773) | 5.8750 (12.3891, 1.1186) |
| Cambodia | 304.2812 (534.3114, 59.4990) | 134.4800 (292.9103, 25.3179) | 1.4270 (3.1289, 0.2700) | 9419.6323 (16074.1162, 1905.3951) | 3482.9818 (7462.1852, 669.7094) | 28.7038 (62.3890, 5.4343) |
| Indonesia | 7949.4705 (13963.7033, 1613.6194) | 9277.7299 (17764.1928, 1834.9594) | 4.4806 (8.4315, 0.8496) | 261992.1582 (448169.1246, 56504.9339) | 278769.8855 (529778.1655, 58187.5339) | 107.1114 (202.7374, 21.7604) |
| Lao People's Democratic Republic | 380.2052 (677.9073, 76.8704) | 127.4924 (257.2046, 22.9872) | 3.2858 (6.7164, 0.5574) | 12002.0931 (21093.7233, 2570.9543) | 3615.4471 (7276.6221, 662.3578) | 71.4896 (144.4206, 13.0427) |
| Malaysia | 283.2610 (562.0041, 53.8102) | 97.0609 (241.0117, 16.3380) | 0.4097 (1.0050, 0.0695) | 7276.3442 (14145.2509, 1455.6012) | 2253.1277 (5707.2427, 386.6412) | 8.1510 (20.2349, 1.4245) |
| Maldives | 0.0363 (0.1182, 0.0025) | 0.0013 (0.0101, 0.0000) | 0.0005 (0.0039, 0.0000) | 1.2114 (3.9306, 0.0659) | 0.0194 (0.1769, 0.0000) | 0.0061 (0.0469, 0.0000) |
| Myanmar | 4006.7875 (7148.5014, 834.7710) | 759.0833 (1600.9996, 123.8927) | 1.9068 (3.8817, 0.3034) | 123883.0011 (217818.1571, 27432.6539) | 18264.9346 (37972.1378, 2970.4800) | 38.5200 (80.0203, 6.3024) |
| Philippines | 1246.7082 (2283.5065, 228.9100) | 1617.3669 (3374.7962, 299.4260) | 2.2154 (4.6705, 0.4151) | 38841.7098 (70424.0311, 7701.3007) | 46591.3574 (96137.8900, 8838.2740) | 52.2860 (108.0545, 9.9390) |
| Sri Lanka | 1053.6000 (1825.1678, 210.1165) | 746.5730 (1479.1962, 126.3304) | 3.0659 (6.1390, 0.5179) | 29798.0942 (50904.1325, 6189.7697) | 17681.0849 (33922.2900, 3053.9700) | 68.2887 (130.6962, 11.8380) |
| Thailand | 1202.1908 (2210.0742, 231.9042) | 703.1027 (1516.5971, 122.1413) | 0.6661 (1.4292, 0.1208) | 32617.2837 (58410.5355, 6506.5929) | 15289.2861 (32290.9489, 2799.0073) | 15.3102 (32.3862, 2.9503) |
| Timor-Leste | 45.5490 (75.1378, 10.9299) | 149.5346 (264.0819, 30.3464) | 19.7451 (34.9879, 3.8593) | 1487.0802 (2426.4984, 370.2039) | 3973.8173 (7091.5865, 876.0012) | 456.7484 (808.6171, 98.3316) |
| Viet Nam | 2138.4185 (3816.0691, 410.8713) | 1326.9403 (2682.7150, 223.2419) | 1.6031 (3.2342, 0.2729) | 54038.0103 (93992.6195, 11049.6412) | 29630.8640 (59886.4237, 4970.2915) | 30.8539 (61.9334, 5.1964) |
| Fiji | 29.6799 (54.3177, 6.0088) | 32.1582 (63.6799, 5.4210) | 5.0742 (10.1186, 0.8676) | 1005.6522 (1872.9366, 213.2134) | 944.8276 (1896.1621, 156.8285) | 118.8821 (233.5282, 19.8227) |
| Kiribati | 0.3651 (0.7854, 0.0583) | 0.6413 (1.5648, 0.1277) | 1.0872 (2.6490, 0.2126) | 11.8340 (26.1308, 1.8798) | 20.7281 (49.0605, 4.2664) | 25.6613 (60.6205, 5.1167) |
| Marshall Islands | 1.2982 (2.4318, 0.2931) | 2.5040 (4.8941, 0.5249) | 7.7250 (15.0875, 1.6249) | 41.7038 (76.7151, 9.7221) | 87.4319 (168.6237, 18.3282) | 202.9660 (390.1704, 42.4410) |
| Micronesia (Federated States of) | 4.6567 (8.7248, 0.8890) | 5.6336 (11.0547, 1.1228) | 8.5506 (16.6868, 1.5983) | 144.7050 (269.3182, 29.2947) | 180.8752 (341.8254, 37.2330) | 219.5845 (424.6528, 43.4997) |
| Papua New Guinea | 183.2579 (321.2742, 37.7710) | 463.0846 (823.9023, 81.4292) | 9.1025 (16.5228, 1.4884) | 6293.9238 (11254.0576, 1321.2457) | 15834.3257 (28475.0933, 2929.9250) | 242.8783 (431.2077, 42.5559) |
| Samoa | 3.8788 (7.3699, 0.7568) | 3.1365 (6.4791, 0.6273) | 2.4898 (5.2549, 0.4702) | 105.8702 (209.7846, 20.4312) | 83.0595 (169.8951, 16.9192) | 56.0886 (114.0279, 11.5246) |
| Solomon Islands | 11.8357 (22.8341, 2.0711) | 32.8137 (62.7648, 6.9187) | 9.9929 (19.2140, 2.0091) | 387.1538 (744.9322, 71.2236) | 1076.2870 (2069.6509, 222.2994) | 254.7327 (489.2944, 53.4352) |
| Tonga | 1.6806 (3.3630, 0.3321) | 1.5034 (3.1981, 0.2700) | 1.9419 (4.0978, 0.3434) | 47.5247 (93.6728, 9.6010) | 36.6615 (79.5495, 6.8028) | 44.7105 (97.6164, 8.1853) |
| Vanuatu | 6.9658 (13.1829, 1.3852) | 19.6074 (37.5415, 4.0809) | 12.1046 (23.1286, 2.4361) | 235.7789 (437.1337, 49.3893) | 646.8615 (1219.9223, 140.6974) | 313.9754 (597.8191, 66.0789) |
| Armenia | 995.0734 (1674.3394, 203.3230) | 1069.2674 (1846.2602, 214.4776) | 25.0194 (43.0860, 5.0408) | 23459.6591 (38659.1571, 5005.9703) | 21472.9404 (36069.1538, 4447.2404) | 511.3203 (852.1803, 106.6957) |
| Azerbaijan | 2088.5053 (3481.6115, 441.6905) | 2921.3264 (5051.4459, 552.7952) | 34.8439 (61.4824, 6.4970) | 53801.1146 (87138.9894, 11918.4558) | 68864.4072 (117712.0748, 13757.8111) | 695.0256 (1195.8977, 134.4138) |
| Georgia | 2517.5648 (4286.9510, 504.0579) | 890.7578 (1560.6623, 163.5190) | 14.5660 (25.3013, 2.7264) | 58073.7546 (97032.7624, 12016.0706) | 19086.8886 (32774.7306, 3707.5132) | 343.7219 (585.0401, 68.1852) |
| Kazakhstan | 4262.4833 (7052.7750, 867.9522) | 3405.6946 (6040.4396, 657.6371) | 23.9439 (42.7493, 4.5085) | 106428.2379 (172245.0488, 22448.2740) | 73892.8247 (128623.1092, 14883.7202) | 442.7483 (779.8237, 87.2535) |
| Kyrgyzstan | 1041.0381 (1720.5244, 220.5662) | 1486.5694 (2487.7634, 317.2465) | 37.6191 (64.1463, 7.8656) | 25746.4068 (42508.0665, 5741.3836) | 34668.5397 (57308.6800, 7713.6163) | 738.4502 (1218.3957, 160.4398) |
| Mongolia | 452.1391 (768.2086, 94.3610) | 561.2565 (928.7712, 118.3058) | 30.6921 (52.1703, 6.2381) | 11090.6224 (18989.4402, 2432.2630) | 14819.2964 (24283.0977, 3268.7976) | 625.2325 (1033.7348, 132.8493) |
| Tajikistan | 1180.5617 (1978.8671, 242.2595) | 1652.4675 (2785.2997, 323.5125) | 35.9721 (61.6772, 6.9160) | 29139.6236 (48342.6177, 6308.3763) | 42346.3553 (70517.3266, 8525.8257) | 730.2537 (1228.0406, 144.1351) |
| Turkmenistan | 752.4297 (1280.4962, 149.5846) | 1035.5014 (1848.5399, 208.1491) | 29.8278 (53.5509, 5.7506) | 19090.8603 (31543.1914, 3914.4517) | 25185.9336 (44926.8591, 5459.7770) | 626.4289 (1109.9651, 130.5282) |
| Uzbekistan | 5020.4608 (8321.6613, 1060.0058) | 10048.7854 (17262.0101, 1950.9742) | 46.3096 (80.2033, 8.7518) | 120473.1455 (193076.4983, 26861.3525) | 250266.3679 (419223.9286, 50662.4507) | 956.9588 (1634.8745, 188.6992) |
| Albania | 404.8492 (672.7134, 82.0139) | 665.7634 (1177.3578, 123.6320) | 16.4343 (29.1420, 3.0672) | 9503.1601 (15233.5771, 2024.2198) | 12189.2264 (21006.0815, 2372.9642) | 297.2302 (510.7155, 58.9758) |
| Bosnia and Herzegovina | 1046.5489 (1749.0698, 216.9646) | 969.6552 (1749.3594, 183.5601) | 15.5162 (27.9105, 2.9554) | 26917.5110 (43817.9020, 5860.7571) | 18434.0469 (32816.0606, 3491.2990) | 307.0394 (545.4213, 59.3704) |
| Bulgaria | 3799.4790 (6574.7419, 758.0945) | 2743.8146 (4769.0931, 519.7704) | 20.3240 (34.8943, 3.9211) | 85863.5112 (144658.8155, 17628.1615) | 54596.4325 (92668.3019, 10836.0130) | 437.0913 (741.6416, 90.4393) |
| Croatia | 1533.7384 (2656.4962, 280.5052) | 1001.7143 (1808.1958, 170.5691) | 10.4372 (18.7532, 1.8136) | 31691.8099 (54058.3594, 6003.9463) | 15972.6603 (28590.7775, 2839.5278) | 182.0814 (321.6844, 33.8891) |
| Czechia | 3941.7753 (6704.6364, 758.7874) | 2194.3593 (3909.0213, 379.9993) | 9.6044 (16.9022, 1.6763) | 85749.2555 (142375.0139, 16859.6216) | 36379.8383 (63826.1365, 6389.4630) | 172.3308 (300.0072, 31.0696) |
| Hungary | 3755.3263 (6444.2944, 714.0138) | 2532.9512 (4399.0698, 456.2237) | 12.2279 (21.0336, 2.2283) | 87988.0285 (148045.7004, 17508.7550) | 45887.0718 (77989.8097, 8479.8214) | 244.5345 (411.8730, 46.7571) |
| North Macedonia | 445.6045 (737.7169, 96.0524) | 506.0579 (886.5172, 91.5399) | 20.5126 (36.4470, 3.7010) | 11181.8356 (17925.1292, 2476.7878) | 10761.3511 (18680.3559, 2030.7950) | 368.2545 (639.1508, 69.5189) |
| Montenegro | 100.3932 (175.1344, 20.0516) | 146.5361 (263.4867, 26.5094) | 17.2700 (31.0556, 3.1647) | 2402.3185 (4039.1444, 494.9317) | 2851.2890 (4988.4321, 545.1681) | 315.5583 (550.8763, 60.7403) |
| Poland | 11227.9096 (19310.9028, 2190.0843) | 5840.5508 (10784.5674, 1077.2716) | 7.6342 (13.9827, 1.4293) | 268539.9074 (449421.0260, 54480.4396) | 102093.1424 (183851.5055, 19487.6357) | 144.5769 (256.8408, 28.0556) |
| Romania | 6986.6114 (11908.5089, 1370.7018) | 5364.4207 (9777.3583, 1009.6931) | 13.7026 (24.8965, 2.5905) | 156207.4632 (259935.7969, 32338.9271) | 99457.3256 (179185.3904, 18689.6408) | 282.5034 (502.6302, 53.7051) |
| Serbia | 2947.1215 (4972.0652, 589.1517) | 2889.6008 (5090.7025, 576.6876) | 17.1334 (30.0251, 3.4590) | 65516.7929 (108762.8267, 13712.6251) | 51300.5236 (89768.8266, 10434.7272) | 321.2233 (559.5967, 66.6486) |
| Slovakia | 2091.7785 (3602.5219, 405.0374) | 1508.6481 (2699.4403, 281.8709) | 16.0720 (28.7659, 3.0284) | 46560.3058 (78439.5137, 9398.6220) | 26945.0739 (46966.9994, 5275.9394) | 291.8127 (504.4961, 58.2080) |
| Slovenia | 326.9427 (571.5592, 63.0575) | 199.7512 (354.0462, 34.1743) | 3.8519 (6.7373, 0.6766) | 7049.2516 (12038.3253, 1391.8520) | 3386.8042 (5888.5937, 616.9692) | 75.9330 (131.0874, 14.3894) |
| Belarus | 3753.6037 (6481.1627, 701.9212) | 3591.4328 (6661.3640, 589.8149) | 22.2032 (41.0713, 3.6918) | 78882.0538 (131789.3978, 15206.1096) | 68135.4880 (124729.7694, 12202.3703) | 437.9876 (795.2960, 79.7726) |
| Estonia | 430.4936 (792.5824, 76.4343) | 109.6575 (213.8121, 17.8478) | 3.0437 (5.8915, 0.4952) | 7885.9238 (14369.9367, 1454.1136) | 1267.9083 (2468.9643, 202.5800) | 36.4941 (70.5195, 5.8882) |
| Latvia | 676.7880 (1218.1735, 118.4387) | 279.3725 (541.4509, 47.7658) | 6.1266 (11.7485, 1.0400) | 13835.7540 (24130.0195, 2504.7689) | 4516.5052 (8571.2405, 770.6492) | 114.0998 (218.0142, 19.6801) |
| Lithuania | 582.5022 (1102.6876, 102.6959) | 180.9124 (383.0507, 31.9846) | 2.5779 (5.4828, 0.4574) | 10875.5459 (20359.4370, 2138.7418) | 2473.2575 (5318.3733, 431.8578) | 39.5850 (87.2098, 7.0534) |
| Republic of Moldova | 1385.5217 (2384.2323, 270.0610) | 1307.5638 (2273.6909, 244.4431) | 21.8364 (37.8669, 4.1116) | 30042.7436 (50622.6528, 6037.8546) | 26620.0344 (45272.1471, 5340.2646) | 457.7206 (775.8140, 92.3672) |
| Russian Federation | 28517.5475 (50113.4282, 5376.0653) | 21865.9100 (40923.7335, 4247.0567) | 9.1982 (17.2354, 1.7931) | 637859.3269 (1104676.4717, 127243.7082) | 427147.8879 (794607.3521, 84994.2137) | 186.2916 (347.0186, 37.4841) |
| Ukraine | 16547.5692 (29687.5692, 2974.3177) | 21600.5386 (39917.0997, 4068.8837) | 27.4649 (50.3945, 5.2018) | 333739.6354 (593865.4319, 62498.5047) | 394089.7586 (721986.4789, 77146.6795) | 519.6919 (947.7112, 102.7591) |
| Brunei Darussalam | 3.1005 (6.1863, 0.6454) | 6.6505 (13.0942, 1.2353) | 2.3594 (4.6783, 0.4204) | 93.4880 (182.4960, 19.6860) | 203.8698 (388.0621, 41.2156) | 51.6328 (99.1435, 9.6433) |
| Japan | 223.9460 (543.1526, 40.9278) | 32.9133 (104.4966, 3.0980) | 0.0043 (0.0140, 0.0005) | 3875.5506 (8898.7460, 763.9297) | 323.3918 (1020.4264, 35.9188) | 0.0548 (0.1701, 0.0066) |
| Republic of Korea | 276.1912 (565.3946, 49.3809) | 107.4321 (272.2858, 16.7969) | 0.1226 (0.3147, 0.0195) | 6883.1532 (13689.6687, 1296.3247) | 1624.5535 (3911.7376, 265.3536) | 1.8861 (4.4558, 0.3197) |
| Singapore | 48.5685 (99.0951, 8.6841) | 3.3876 (9.8729, 0.3648) | 0.0415 (0.1201, 0.0044) | 1276.8367 (2548.7811, 241.4800) | 73.7566 (211.4777, 9.1538) | 0.8868 (2.5393, 0.1106) |
| Australia | 2062.8798 (3659.8565, 357.1656) | 892.1462 (1700.1968, 160.4773) | 1.7316 (3.2268, 0.3223) | 41048.9863 (70172.0784, 7350.9135) | 14296.3537 (26151.5836, 2715.7206) | 32.1815 (58.8700, 6.2042) |
| New Zealand | 438.3517 (796.3196, 78.9118) | 214.6162 (402.4758, 38.6249) | 2.3700 (4.4201, 0.4348) | 9225.8372 (16343.2695, 1719.8427) | 3574.9108 (6446.4136, 690.0915) | 43.6043 (77.0691, 8.6608) |
| Andorra | 3.5594 (6.4681, 0.6576) | 4.1677 (7.6557, 0.7181) | 2.3970 (4.3944, 0.4141) | 82.9802 (149.4559, 16.0344) | 72.9254 (131.3454, 13.3508) | 46.0191 (83.5506, 8.5986) |
| Austria | 1758.0506 (3117.8707, 336.3353) | 861.0559 (1625.5348, 148.5844) | 3.7918 (7.1465, 0.6606) | 33470.6291 (57767.4202, 6467.2423) | 12194.1887 (22581.4282, 2181.1794) | 62.2608 (113.7268, 11.5206) |
| Belgium | 1051.6993 (1914.7812, 178.0509) | 347.0831 (687.0537, 61.1951) | 1.2251 (2.3884, 0.2225) | 19837.4677 (35949.7793, 3488.5231) | 5487.2245 (10862.0555, 1018.9961) | 23.3787 (45.9751, 4.4389) |
| Cyprus | 111.9960 (198.3746, 20.4540) | 77.2094 (146.1198, 13.4248) | 4.6630 (8.9452, 0.7934) | 2256.2106 (3910.9118, 420.6590) | 1447.5227 (2682.3896, 253.4746) | 79.4400 (148.5303, 14.0841) |
| Denmark | 902.5420 (1694.7113, 161.0927) | 183.7047 (355.0668, 33.0617) | 1.3915 (2.6646, 0.2529) | 16086.4879 (29452.9659, 2995.6816) | 2970.1251 (5620.3566, 540.3711) | 25.9433 (48.6715, 4.9217) |
| Finland | 421.3575 (852.8021, 68.2856) | 184.5680 (394.5721, 29.5333) | 1.0873 (2.3020, 0.1727) | 7722.5464 (15422.7001, 1334.1573) | 2277.6950 (4785.5638, 356.7983) | 15.1406 (31.1885, 2.4062) |
| France | 2414.9080 (4733.5687, 430.5637) | 1193.1139 (2453.7252, 201.7758) | 0.6168 (1.2649, 0.1076) | 40996.7015 (77642.0671, 7715.4175) | 17186.1948 (35050.5731, 3033.9841) | 11.4496 (23.0497, 2.0638) |
| Germany | 20371.8923 (36362.2445, 3822.4685) | 8760.6662 (16223.4314, 1684.3975) | 3.8214 (7.0576, 0.7445) | 386045.5736 (673341.2608, 75675.9757) | 138070.9826 (248668.7314, 26924.3332) | 71.6931 (126.4468, 14.2842) |
| Greece | 1415.0552 (2538.9506, 246.4624) | 1277.2763 (2326.1869, 236.3772) | 4.6259 (8.3590, 0.8945) | 29080.5961 (50638.3804, 5302.1658) | 21936.0052 (39486.3696, 4319.1864) | 102.8079 (181.5569, 20.6811) |
| Iceland | 2.0358 (4.8433, 0.2919) | 0.8904 (2.3650, 0.1298) | 0.1208 (0.3278, 0.0170) | 29.6256 (69.2736, 4.6492) | 10.7881 (29.4010, 1.6268) | 1.6589 (4.2652, 0.2543) |
| Ireland | 582.0846 (1039.2162, 110.1439) | 105.6549 (206.2367, 20.1605) | 1.2542 (2.4374, 0.2382) | 11861.3140 (20827.2998, 2237.8776) | 1667.6114 (3167.8168, 303.4163) | 21.1922 (41.1634, 3.8759) |
| Israel | 379.3586 (683.7520, 66.6249) | 143.3985 (285.2910, 25.3267) | 1.0253 (2.0289, 0.1860) | 7502.2369 (13338.4047, 1332.6960) | 2268.8860 (4337.1131, 426.2736) | 17.9284 (34.7575, 3.4090) |
| Italy | 5658.3227 (10129.8648, 1034.6899) | 3945.7184 (7339.8239, 766.0399) | 2.0968 (3.7985, 0.4123) | 110701.0426 (191800.9118, 21155.4987) | 57299.5373 (103228.5181, 11075.2909) | 38.0025 (67.9445, 7.3609) |
| Luxembourg | 40.6908 (74.6724, 7.2878) | 15.4543 (29.8994, 2.8253) | 1.2684 (2.4459, 0.2330) | 790.0474 (1422.1949, 153.1127) | 244.2513 (468.2528, 44.5925) | 22.0439 (41.6527, 3.9811) |
| Malta | 53.5148 (93.7183, 9.2682) | 20.6068 (41.2902, 3.4738) | 1.8800 (3.7138, 0.3171) | 1102.2715 (1921.6575, 201.8772) | 320.6873 (631.5520, 54.1430) | 33.6835 (65.9758, 5.6935) |
| Netherlands | 1721.6260 (3138.8869, 308.3580) | 580.0819 (1134.1484, 95.8123) | 1.4673 (2.8584, 0.2461) | 34168.9947 (61880.6145, 6247.4534) | 8818.5221 (16770.3015, 1540.6403) | 24.7088 (47.1091, 4.4214) |
| Norway | 353.6264 (686.1994, 61.0868) | 67.8798 (144.5175, 12.0467) | 0.5561 (1.1852, 0.1006) | 6266.0148 (12005.4836, 1144.2010) | 969.2936 (2032.7061, 182.4151) | 9.0994 (19.0282, 1.7631) |
| Portugal | 375.5795 (743.7081, 63.3163) | 162.7763 (340.9879, 29.2278) | 0.5425 (1.1363, 0.0980) | 7023.9292 (13444.7359, 1213.8344) | 2493.7321 (5241.2236, 446.5821) | 10.2592 (21.9092, 1.8735) |
| Spain | 1553.1184 (3073.8912, 284.7446) | 709.3722 (1525.8272, 125.8158) | 0.5582 (1.1858, 0.1015) | 28345.0798 (53767.4546, 5135.3109) | 10650.3100 (22312.6572, 1954.8282) | 10.4652 (21.5010, 2.0036) |
| Sweden | 1247.1442 (2319.6470, 213.0165) | 361.3570 (765.1156, 60.9512) | 1.2921 (2.6769, 0.2205) | 20540.2588 (38038.3451, 3520.7806) | 4831.2786 (10019.9561, 844.1116) | 20.0361 (41.6683, 3.7277) |
| Switzerland | 1109.5709 (2027.5158, 209.8071) | 466.9831 (879.5490, 83.7321) | 1.9805 (3.7185, 0.3615) | 20165.5273 (35701.1397, 3831.2383) | 6618.1222 (12214.8156, 1263.9794) | 33.6038 (60.7800, 6.6546) |
| United Kingdom | 9705.9342 (17448.4539, 1871.7341) | 2766.6148 (5436.2381, 557.6056) | 1.9384 (3.7332, 0.3917) | 190703.7638 (338298.2245, 37449.9702) | 48534.8916 (91086.4928, 9828.1964) | 39.6708 (73.7222, 8.0952) |
| Argentina | 5103.2004 (8838.6838, 1003.7843) | 3135.5234 (5465.0025, 596.1048) | 5.5071 (9.5855, 1.0515) | 117103.2231 (198651.3441, 23561.1690) | 65495.5661 (114485.0811, 12729.6261) | 119.5548 (208.3653, 23.3536) |
| Chile | 837.5896 (1471.6298, 157.8929) | 573.3976 (1042.5645, 103.1246) | 2.2243 (4.0321, 0.4028) | 17980.6499 (31248.5872, 3519.7352) | 12700.9791 (22624.9589, 2442.5326) | 51.0148 (90.2251, 9.8996) |
| Uruguay | 691.9464 (1177.0972, 136.1037) | 347.3750 (611.1093, 61.5423) | 5.7756 (10.0336, 1.0436) | 14817.0166 (24768.3195, 2999.0528) | 6671.4635 (11398.4604, 1240.2236) | 128.1408 (215.2532, 24.5208) |
| Canada | 2198.6568 (4161.2451, 388.6394) | 1400.4270 (2724.6967, 251.4810) | 1.7498 (3.4000, 0.3239) | 42591.3059 (78332.2969, 7655.4117) | 23200.1257 (44419.8832, 4389.1550) | 33.2461 (62.7643, 6.5053) |
| United States of America | 41748.3728 (74730.7716, 7683.2522) | 36886.2055 (66671.9707, 6932.2525) | 6.1602 (11.0136, 1.1886) | 814774.9094 (1414244.4302, 153882.8352) | 737234.6632 (1282830.8303, 144967.3401) | 137.1569 (234.5428, 27.4178) |
| Antigua and Barbuda | 2.2189 (4.1993, 0.3895) | 0.9552 (1.9322, 0.1465) | 1.0748 (2.2051, 0.1622) | 43.5742 (82.8598, 7.9035) | 18.9404 (38.6390, 2.9867) | 18.9977 (38.7658, 2.9756) |
| Bahamas | 10.5441 (18.7699, 1.9658) | 12.4591 (23.4923, 2.4044) | 3.3215 (6.2266, 0.6349) | 295.2558 (518.5318, 57.9483) | 321.5178 (610.7565, 64.3938) | 76.7409 (145.6282, 15.2301) |
| Barbados | 14.5416 (28.3144, 2.6258) | 11.4534 (21.6143, 1.9958) | 2.2389 (4.2103, 0.3915) | 270.1886 (512.3431, 49.3482) | 214.2464 (396.8677, 38.5152) | 43.1871 (79.9867, 7.8296) |
| Belize | 9.9523 (17.2068, 1.8517) | 12.9922 (23.1443, 2.4434) | 4.6216 (8.3202, 0.8401) | 239.8148 (401.4470, 47.0981) | 335.1536 (572.6466, 66.7998) | 105.2878 (184.4722, 20.5038) |
| Cuba | 1625.4206 (2822.6106, 292.6723) | 1553.8451 (2799.6397, 281.2412) | 7.5657 (13.6120, 1.3818) | 35563.4519 (60987.2160, 6650.6309) | 31012.3250 (54270.1268, 5771.3866) | 161.7104 (279.7814, 30.4765) |
| Dominica | 5.2840 (9.3864, 0.9291) | 3.1283 (6.1122, 0.5546) | 4.0802 (8.0058, 0.7291) | 105.9731 (189.5495, 19.8460) | 65.0297 (122.9558, 12.1078) | 80.3873 (153.3935, 15.1286) |
| Dominican Republic | 558.0833 (955.7574, 111.1508) | 1098.5020 (2016.5833, 218.3002) | 11.0716 (20.4463, 2.1630) | 15541.2958 (25739.1667, 3251.4913) | 27991.8343 (51663.9163, 5917.5085) | 270.1559 (496.5636, 56.9918) |
| Grenada | 6.0114 (10.7552, 1.0654) | 2.7071 (5.5129, 0.4676) | 2.7879 (5.6207, 0.4681) | 134.3061 (237.0440, 25.0684) | 62.6120 (124.1159, 11.1715) | 56.9066 (111.7793, 9.9507) |
| Guyana | 46.6298 (85.1438, 8.9666) | 26.4519 (52.5644, 4.2498) | 4.6222 (9.0296, 0.7532) | 1309.4679 (2348.3940, 268.6002) | 692.4448 (1404.5838, 112.0870) | 105.1447 (209.2828, 16.9746) |
| Haiti | 1116.8606 (1875.7815, 226.7111) | 1933.8912 (3392.3476, 444.4174) | 29.7530 (53.2313, 6.5265) | 32343.5342 (53412.2355, 6840.4968) | 56282.0192 (97216.0252, 13525.3200) | 705.4681 (1232.8177, 163.2073) |
| Jamaica | 81.0466 (144.9375, 14.9428) | 90.5501 (169.6949, 16.1771) | 2.6982 (5.0338, 0.4880) | 1652.7934 (2855.0893, 314.0429) | 1870.2434 (3415.4383, 361.3461) | 59.0071 (107.4336, 11.4741) |
| Saint Lucia | 6.5692 (11.9752, 1.1325) | 4.0937 (7.7536, 0.6868) | 1.7897 (3.3977, 0.3027) | 143.3828 (251.3842, 25.8849) | 85.3852 (159.6469, 15.5755) | 36.2094 (67.6897, 6.5958) |
| Saint Vincent and the Grenadines | 10.9803 (19.2607, 2.1240) | 8.3085 (15.2616, 1.5009) | 6.5766 (12.0240, 1.1748) | 252.8002 (431.7610, 50.9908) | 180.2338 (325.0124, 34.0377) | 132.8647 (238.5817, 24.9354) |
| Suriname | 42.3668 (72.2085, 8.0306) | 45.8278 (84.5257, 9.7023) | 7.3877 (13.5980, 1.5587) | 1127.3966 (1884.4209, 224.4670) | 1207.9070 (2202.7123, 272.2995) | 185.9860 (337.9554, 41.6604) |
| Trinidad and Tobago | 156.5352 (269.7054, 29.7409) | 112.7675 (210.2019, 20.3191) | 6.0722 (11.3940, 1.0931) | 4029.6948 (6862.8055, 796.1003) | 2712.4616 (5004.6975, 510.2292) | 145.4385 (267.2374, 27.4678) |
| Bolivia (Plurinational State of) | 558.7970 (1015.2398, 101.2702) | 789.7682 (1488.5363, 139.9006) | 9.8830 (18.6940, 1.7381) | 15164.6980 (27457.8284, 2972.3090) | 19443.4274 (35551.7699, 3565.1828) | 211.4026 (391.3771, 38.3550) |
| Ecuador | 536.9773 (906.0694, 107.9585) | 1143.2258 (2036.4900, 225.2660) | 7.5885 (13.5269, 1.4836) | 13755.9793 (22382.5680, 2952.1466) | 26255.3214 (45279.9187, 5447.6751) | 160.9958 (279.1359, 33.1185) |
| Peru | 538.0381 (939.8274, 102.8246) | 542.4107 (1083.4117, 95.3162) | 1.5880 (3.1894, 0.2751) | 13700.0891 (23553.9732, 2685.9725) | 12315.6309 (23940.3946, 2366.9067) | 35.2474 (69.0777, 6.6719) |
| Colombia | 2825.0217 (4689.4553, 567.1688) | 4168.1691 (7431.3313, 740.0249) | 7.3543 (13.0918, 1.3065) | 74786.2797 (122149.6209, 15776.1406) | 87011.3398 (150723.4682, 16137.5289) | 156.4691 (270.3103, 29.1087) |
| Costa Rica | 233.2779 (404.6830, 47.2735) | 256.4113 (448.3035, 47.3703) | 4.5487 (7.9373, 0.8474) | 5402.4902 (9028.7280, 1168.0980) | 6001.3831 (10112.9693, 1204.8008) | 108.5121 (182.8367, 21.8926) |
| El Salvador | 479.9563 (802.4135, 96.6946) | 715.8442 (1277.4873, 135.1768) | 10.8157 (19.2706, 2.0513) | 12461.5367 (19974.9924, 2645.7785) | 15780.7222 (27554.6528, 3263.2463) | 251.8284 (439.3728, 52.4570) |
| Guatemala | 590.7925 (967.0239, 124.0650) | 1139.5220 (1952.9072, 232.0362) | 11.8946 (20.6302, 2.3896) | 17137.0505 (27466.1752, 3827.6958) | 27575.7727 (45485.5303, 5751.5856) | 246.7222 (413.0018, 50.7974) |
| Honduras | 280.8591 (480.8427, 56.8995) | 1067.8397 (1854.4050, 214.5754) | 19.8250 (34.3799, 3.8695) | 7739.2964 (13072.6224, 1669.7281) | 25713.2869 (44151.6600, 5463.2800) | 408.1726 (706.0678, 84.6562) |
| Mexico | 3610.0405 (6183.7042, 714.6093) | 10164.1502 (18352.4896, 1960.1293) | 8.6923 (15.7769, 1.6420) | 89945.7758 (150669.2278, 18334.6574) | 235150.2995 (408802.2920, 48384.7193) | 184.6972 (323.2974, 37.6047) |
| Nicaragua | 177.2813 (295.4880, 38.1959) | 453.6885 (769.7427, 98.5360) | 10.3548 (17.7469, 2.1776) | 4680.2121 (7624.2183, 1069.1580) | 11127.1260 (18336.5313, 2550.2612) | 221.2023 (366.7414, 49.8010) |
| Panama | 138.2248 (238.8874, 25.9204) | 155.0862 (278.8257, 28.4013) | 3.3924 (6.0929, 0.6227) | 3145.5094 (5239.4712, 626.2637) | 3418.9890 (5936.2938, 652.8804) | 76.6102 (133.0112, 14.6191) |
| Venezuela (Bolivarian Republic of) | 1152.6564 (2018.1725, 221.2383) | 3334.9859 (6060.1857, 629.3819) | 11.4930 (20.8577, 2.1411) | 31194.5710 (53445.5414, 6295.5511) | 81974.8909 (146163.9863, 16981.4667) | 270.1094 (481.9875, 55.4675) |
| Brazil | 12393.0209 (20884.8275, 2455.0484) | 14488.8880 (25225.1221, 2807.3082) | 5.8159 (10.1261, 1.1293) | 353621.6535 (582784.9384, 74191.2973) | 385223.4655 (646961.1509, 75917.8624) | 150.8830 (254.0896, 29.7222) |
| Paraguay | 252.2514 (433.5199, 47.7700) | 429.2232 (780.3878, 80.6086) | 7.6161 (13.8689, 1.4118) | 6368.4598 (10701.6870, 1297.1469) | 10603.0816 (19016.6391, 2093.3562) | 176.1560 (316.5896, 34.2929) |
| Algeria | 3550.7213 (6093.3436, 734.2235) | 6752.6708 (12023.6519, 1359.9789) | 24.7033 (44.3860, 4.9079) | 98028.4724 (162723.5840, 21531.4568) | 162014.5833 (282353.9052, 34863.3509) | 467.6898 (819.0627, 97.1161) |
| Bahrain | 45.3136 (77.0135, 9.2517) | 59.6142 (107.3981, 11.3113) | 9.7943 (18.1403, 1.7266) | 1403.8172 (2335.4453, 307.7873) | 1857.5475 (3248.4905, 370.5223) | 186.1114 (336.9525, 34.0006) |
| Egypt | 8319.1933 (13756.3442, 1762.4808) | 9080.2252 (16133.8671, 1679.1874) | 18.0840 (32.5086, 3.3183) | 251354.6915 (408468.4784, 55335.8260) | 262320.0399 (459137.6412, 50704.4049) | 393.2670 (693.2994, 73.0016) |
| Iran (Islamic Republic of) | 7211.0427 (11829.2503, 1500.5939) | 10073.3587 (17597.9500, 1964.8026) | 14.2207 (25.2965, 2.7469) | 205077.9536 (329001.5122, 44281.5915) | 242572.8041 (411396.8490, 50015.0152) | 300.0323 (514.5719, 59.9113) |
| Iraq | 2820.7590 (4794.9073, 567.7496) | 6163.2363 (10882.3511, 1220.9166) | 30.1076 (52.4730, 5.7830) | 77123.0092 (127410.3728, 16061.9502) | 165428.8126 (291814.3136, 35043.4484) | 654.3219 (1150.0080, 131.5432) |
| Jordan | 309.8392 (524.3548, 67.6634) | 706.7181 (1229.4110, 139.9524) | 10.8450 (19.1657, 2.0230) | 9407.5396 (15631.1420, 2116.9007) | 20732.4297 (35325.9452, 4359.4607) | 246.0197 (425.5439, 49.3768) |
| Kuwait | 116.3556 (198.6734, 23.4561) | 228.2040 (409.2175, 43.0918) | 7.2925 (13.5228, 1.2413) | 3884.3241 (6379.4682, 820.1583) | 7415.8608 (13319.7782, 1503.7232) | 175.2083 (311.2132, 32.4407) |
| Lebanon | 576.4424 (1012.6149, 117.1184) | 575.9500 (1023.0916, 117.7881) | 8.9043 (15.7732, 1.8306) | 15698.6987 (26827.2309, 3285.5429) | 11783.9388 (20276.0805, 2514.3852) | 191.3815 (328.8059, 40.7955) |
| Libya | 282.0839 (493.3096, 56.1868) | 952.4337 (1684.2917, 221.7874) | 18.7155 (33.7821, 4.0244) | 7902.0311 (13571.8358, 1665.0594) | 28096.3564 (49086.9774, 6881.1421) | 452.7445 (795.1872, 106.7648) |
| Morocco | 4879.7257 (8500.2150, 1010.7434) | 7585.5992 (13854.3043, 1556.9661) | 24.5588 (44.8495, 4.9480) | 134219.0157 (228184.4181, 29015.9325) | 188782.9619 (337102.1956, 40790.3338) | 540.7790 (979.4285, 114.6602) |
| Palestine | 305.2082 (533.0050, 58.0868) | 498.9558 (813.2039, 110.9547) | 23.9077 (39.7147, 5.0729) | 7634.2032 (13062.2013, 1579.7004) | 13297.7685 (21269.4298, 3117.1777) | 496.7618 (806.2286, 111.3688) |
| Oman | 113.0696 (203.8888, 21.5955) | 131.7769 (245.8258, 26.1271) | 8.0672 (15.0883, 1.5387) | 3257.8675 (5959.6621, 653.2999) | 3894.0646 (7183.5471, 814.0174) | 163.4640 (305.5254, 31.4120) |
| Qatar | 16.2086 (30.0984, 3.2061) | 19.6821 (39.7806, 3.8553) | 3.2091 (6.5454, 0.5264) | 538.1244 (968.9183, 108.2395) | 696.4189 (1375.1097, 138.7412) | 56.5239 (117.1177, 9.5524) |
| Saudi Arabia | 1376.7797 (2382.5810, 277.7041) | 3468.4842 (6093.0686, 681.7457) | 15.7212 (28.4328, 2.8686) | 42290.0837 (72771.1521, 8790.9132) | 128394.5279 (226670.1207, 25901.0749) | 409.2795 (735.9443, 78.5176) |
| Syrian Arab Republic | 2675.1437 (4444.0472, 639.9482) | 5424.4812 (9326.1294, 1219.4031) | 48.9939 (84.4743, 10.4877) | 78744.6837 (128830.4410, 19555.4026) | 143430.0127 (246559.0157, 34288.2887) | 1072.3803 (1832.7431, 248.4675) |
| Tunisia | 1030.5263 (1775.8121, 193.5710) | 1731.2566 (3254.5574, 313.9887) | 14.3166 (26.9311, 2.5568) | 26836.3255 (45156.9910, 5230.2039) | 39475.2218 (72856.5142, 7709.9629) | 300.2454 (551.9719, 58.0599) |
| Turkey | 6938.5582 (11955.4737, 1488.3632) | 7149.6233 (12942.4714, 1482.8478) | 8.4357 (15.2435, 1.7379) | 189743.9329 (317927.2297, 42442.0945) | 151470.4243 (269045.8489, 32525.5049) | 167.3044 (296.4864, 35.7697) |
| United Arab Emirates | 54.9609 (103.6065, 10.7486) | 201.3638 (356.8985, 38.4477) | 9.0732 (16.8460, 1.6158) | 1859.2078 (3413.9643, 374.8580) | 7177.0621 (12512.7976, 1388.1252) | 168.0811 (309.9255, 30.7773) |
| Yemen | 2029.1807 (3600.9012, 416.4504) | 4734.9799 (8465.3726, 1070.4849) | 36.4008 (65.0685, 7.9127) | 61854.2952 (110954.2406, 13500.1050) | 139023.6478 (242649.8340, 32654.5679) | 862.2767 (1536.6933, 196.1623) |
| Afghanistan | 4129.8900 (7166.2497, 814.2705) | 4584.4089 (7833.7277, 1036.7776) | 46.3304 (80.2316, 9.8257) | 119343.4303 (204544.6761, 24143.9712) | 150423.5632 (255073.2447, 35066.7148) | 1178.8903 (2021.1134, 262.7177) |
| Bangladesh | 5007.2230 (8606.7308, 1013.6520) | 3838.9054 (7511.2712, 719.1053) | 2.9820 (5.7953, 0.5540) | 161110.5426 (274028.8771, 34599.9149) | 103944.9140 (202248.7222, 19632.5102) | 72.2457 (139.9191, 13.5406) |
| Bhutan | 37.5298 (63.6625, 7.9396) | 62.8426 (111.6623, 11.3343) | 10.7311 (19.2328, 1.8859) | 1223.6125 (2090.3198, 270.2436) | 1636.6890 (2846.1648, 320.4566) | 254.3701 (446.1015, 48.8599) |
| India | 92440.0897 (152257.6283, 20569.3755) | 176366.4299 (296207.8054, 34857.9578) | 15.4386 (26.0917, 3.0053) | 3017565.1394 (4871465.4785, 697075.1836) | 5066234.3648 (8314562.1388, 1041339.9605) | 398.3170 (657.5238, 80.8938) |
| Nepal | 1956.7228 (3208.5507, 465.8380) | 4060.3660 (7074.7382, 831.4699) | 18.8770 (33.3717, 3.7218) | 62047.6051 (100878.7282, 15281.0807) | 111936.8418 (188096.8743, 24360.1869) | 459.8837 (784.5696, 97.7078) |
| Pakistan | 11542.9946 (20346.0149, 2494.7887) | 29989.8150 (52583.6022, 6481.1770) | 25.2187 (44.5667, 5.2639) | 338413.0650 (579183.9967, 76131.3535) | 942733.1222 (1619260.2334, 210904.6439) | 646.2194 (1129.1019, 140.5540) |
| Angola | 360.1552 (632.8636, 76.3045) | 824.3087 (1521.0611, 144.9606) | 8.4731 (15.8991, 1.5171) | 10742.4241 (18965.4782, 2320.6980) | 23812.7504 (42704.9195, 4148.4745) | 187.5129 (346.5149, 33.0735) |
| Central African Republic | 176.4493 (323.9583, 34.3848) | 315.6064 (577.5361, 64.4541) | 16.1259 (28.9693, 3.1482) | 5314.8228 (9674.7769, 1072.3683) | 9963.3127 (18372.4113, 2041.3793) | 389.2034 (707.5332, 78.5992) |
| Congo | 45.9650 (92.4909, 8.5797) | 114.6860 (221.3089, 21.0701) | 5.2110 (10.0263, 0.9121) | 1284.7454 (2566.9069, 254.0709) | 3283.2740 (6410.1889, 661.8078) | 112.4140 (214.8618, 20.6398) |
| Democratic Republic of the Congo | 1552.1675 (2930.0112, 300.0923) | 3650.8040 (6725.0944, 668.4538) | 11.6973 (21.8641, 2.1203) | 44097.2407 (83306.3335, 8881.8619) | 104348.8001 (190903.0954, 19306.1834) | 264.5758 (489.5514, 48.4836) |
| Equatorial Guinea | 24.3263 (43.7729, 4.7187) | 4.3103 (10.4763, 0.7790) | 1.1077 (2.5681, 0.1718) | 696.1006 (1255.4174, 139.5401) | 112.9889 (286.0060, 20.3033) | 21.4288 (51.8718, 3.9045) |
| Gabon | 13.2538 (29.8711, 2.1979) | 18.1887 (38.9570, 3.3646) | 2.2595 (4.7965, 0.3990) | 309.6504 (684.8299, 53.5494) | 449.6438 (958.6330, 87.3572) | 44.2351 (94.4665, 8.2892) |
| Burundi | 323.4675 (563.4717, 66.7864) | 558.1501 (963.2998, 110.4765) | 12.3401 (21.8433, 2.3035) | 9447.3195 (16198.8131, 2095.1190) | 17242.5477 (29408.4865, 3663.8741) | 300.7659 (521.6958, 59.5792) |
| Comoros | 7.2534 (13.3772, 1.4567) | 21.8221 (40.4447, 3.7557) | 4.9326 (9.1079, 0.8392) | 213.6063 (388.1990, 43.4085) | 598.3072 (1097.4988, 107.2006) | 114.6486 (212.4618, 19.9232) |
| Djibouti | 13.0140 (22.7720, 2.8621) | 75.3619 (138.1748, 17.3355) | 13.1397 (23.6599, 2.8614) | 424.5953 (743.1084, 95.7560) | 2377.6963 (4330.9812, 569.2883) | 314.3554 (571.1789, 71.5526) |
| Eritrea | 125.0711 (215.7618, 27.4548) | 286.4115 (500.6398, 64.4799) | 11.1768 (19.5701, 2.4075) | 4296.3205 (7367.3560, 961.5478) | 9385.0220 (16498.5212, 2188.4727) | 276.2368 (480.2296, 61.9076) |
| Ethiopia | 2649.9682 (4479.6870, 599.6519) | 3471.6029 (5762.4006, 748.5962) | 8.4826 (14.3586, 1.7697) | 84158.7868 (140263.3299, 19896.9990) | 100600.1707 (165689.2402, 22501.1058) | 203.5853 (337.9859, 44.1631) |
| Kenya | 305.4546 (538.6565, 65.8086) | 1190.8600 (2082.2945, 246.2360) | 6.0804 (10.7816, 1.2189) | 8547.4607 (14778.4910, 1911.4943) | 34414.9079 (59454.2823, 7505.0052) | 138.1206 (241.1814, 28.8360) |
| Madagascar | 468.4470 (790.1200, 100.8426) | 1298.1408 (2295.8280, 275.0576) | 12.2296 (22.2294, 2.3790) | 14384.4194 (23851.2496, 3255.2497) | 42811.0721 (74282.7756, 9363.1896) | 307.7978 (545.8571, 64.1996) |
| Malawi | 231.4456 (402.0119, 46.2786) | 577.4414 (988.2537, 111.6305) | 8.2857 (14.4029, 1.6074) | 7017.0440 (12106.0843, 1450.9728) | 17912.8358 (30934.1752, 3654.1714) | 206.3544 (353.3889, 39.8847) |
| Mauritius | 130.4663 (225.6499, 24.3289) | 78.2161 (142.4088, 14.4412) | 4.6551 (8.5148, 0.8603) | 3744.0328 (6361.4272, 724.6721) | 1904.1552 (3395.8928, 366.8399) | 112.1491 (198.2702, 21.7833) |
| Mozambique | 259.4920 (434.9927, 53.8853) | 663.1113 (1137.9455, 134.1819) | 6.6236 (11.4783, 1.2796) | 7512.0901 (12309.0283, 1635.9101) | 20634.1565 (35229.3601, 4388.6611) | 162.5971 (278.6661, 33.2881) |
| Rwanda | 412.8909 (732.1320, 90.8954) | 503.0581 (919.4395, 108.4161) | 9.2203 (16.8146, 1.8807) | 12589.2647 (22125.5866, 2819.4739) | 14331.7573 (25680.5439, 3177.5025) | 209.6270 (379.4523, 45.5679) |
| Seychelles | 1.1026 (2.2148, 0.2038) | 0.4224 (0.9635, 0.0686) | 0.4281 (0.9686, 0.0707) | 25.8916 (51.0983, 5.1311) | 9.6477 (22.0981, 1.6037) | 8.6210 (19.5585, 1.4009) |
| Somalia | 244.8753 (448.1995, 52.0757) | 644.7464 (1178.3907, 140.7902) | 11.0494 (20.5669, 2.3462) | 8224.6649 (15144.5524, 1785.2853) | 21576.6129 (39438.6705, 4818.6251) | 282.3537 (513.0320, 60.5933) |
| United Republic of Tanzania | 490.7236 (882.9770, 100.3067) | 1421.6671 (2639.1697, 265.0522) | 6.0032 (11.2915, 1.0799) | 14134.0030 (25113.1955, 2975.5904) | 40780.8467 (74976.0353, 8100.7616) | 141.8151 (261.1420, 26.6059) |
| Uganda | 265.9089 (493.7350, 51.7454) | 428.3536 (814.1747, 75.4835) | 3.3158 (6.3072, 0.5947) | 7355.9923 (13366.3520, 1493.3948) | 12342.2345 (23476.8664, 2250.7714) | 74.4848 (141.8684, 13.2936) |
| Zambia | 126.0446 (218.9398, 25.2524) | 376.3752 (706.2249, 80.8023) | 6.0958 (11.4147, 1.2350) | 3720.5482 (6394.7688, 787.6302) | 11277.8128 (21216.4433, 2504.6556) | 140.6662 (264.4959, 30.1109) |
| Botswana | 68.8960 (119.2157, 13.9378) | 126.4567 (220.4048, 27.4832) | 9.8630 (17.6782, 2.0992) | 1980.3376 (3416.9282, 426.5231) | 3554.0623 (6142.0595, 802.7102) | 223.7714 (391.1488, 48.6538) |
| Lesotho | 60.3049 (100.0726, 13.0862) | 140.6090 (265.7668, 31.2669) | 14.4887 (26.6947, 3.0883) | 1509.8659 (2510.8111, 338.1488) | 4044.2266 (7677.4471, 931.5286) | 354.5342 (670.2620, 79.7386) |
| Namibia | 63.6514 (114.4075, 13.6551) | 109.6024 (195.3086, 22.7097) | 9.1891 (16.2465, 1.8831) | 1774.4208 (3135.0921, 390.0104) | 3002.6061 (5369.7650, 642.8967) | 206.4450 (367.6641, 43.2583) |
| South Africa | 1674.1168 (2889.3117, 366.3895) | 3307.7705 (5719.8269, 673.7323) | 7.9544 (13.9055, 1.5884) | 51171.8983 (84209.3016, 11746.6075) | 89490.3701 (151382.5740, 19356.3858) | 184.0677 (313.4769, 38.7654) |
| Eswatini | 34.4263 (58.6679, 7.2313) | 77.2429 (144.9523, 16.6754) | 15.0086 (28.1827, 3.0966) | 990.1021 (1673.2879, 216.0432) | 2384.2608 (4474.8614, 533.7418) | 374.4294 (697.9030, 80.6160) |
| Zimbabwe | 350.3091 (607.3600, 72.9724) | 999.8301 (1712.4034, 211.6126) | 16.9813 (29.6270, 3.4527) | 8847.3067 (15152.4342, 1903.7589) | 29198.6433 (50099.9310, 6480.9841) | 391.4959 (667.1562, 82.9677) |
| Benin | 118.7150 (216.2495, 24.1204) | 283.8037 (510.4336, 53.2675) | 6.3465 (11.5962, 1.1649) | 2968.9484 (5220.3941, 641.1439) | 7468.4035 (13160.3563, 1488.1377) | 137.2678 (244.8442, 26.1578) |
| Burkina Faso | 451.4196 (780.6748, 94.6993) | 991.8731 (1761.9879, 183.7206) | 12.4090 (22.3448, 2.2963) | 11768.6963 (20065.2289, 2609.6211) | 25051.8106 (43849.6855, 4845.2262) | 266.5394 (464.2197, 49.6795) |
| Cameroon | 259.9134 (466.8528, 51.4547) | 824.8675 (1528.7405, 172.2795) | 7.7339 (14.2642, 1.6114) | 6930.8625 (12370.7361, 1399.4153) | 22758.3652 (41810.4118, 4868.0681) | 168.8600 (313.0864, 35.4117) |
| Cabo Verde | 15.3678 (27.7307, 2.8973) | 29.1017 (55.5676, 5.2108) | 6.8696 (13.1045, 1.2140) | 339.1413 (597.2936, 67.6649) | 621.5276 (1177.0863, 115.1662) | 137.1557 (262.6135, 24.6773) |
| Chad | 243.2032 (438.9168, 51.1878) | 460.8080 (805.0679, 98.0337) | 9.0923 (15.9360, 1.8482) | 6081.7147 (10796.2612, 1354.9747) | 12773.5338 (22495.7897, 2813.0958) | 206.4217 (359.4281, 44.0663) |
| C么te d'Ivoire | 299.7169 (557.4339, 59.5006) | 869.5408 (1578.3629, 188.6021) | 9.1314 (16.6134, 1.8683) | 8831.4518 (16066.2433, 1801.0691) | 24973.9390 (45456.6154, 5768.5601) | 202.1364 (365.7028, 43.7792) |
| Gambia | 19.3299 (35.4185, 3.7277) | 66.5355 (125.3461, 11.1810) | 7.7808 (14.5971, 1.2913) | 522.1700 (965.2018, 107.8381) | 1722.3930 (3219.0728, 297.3017) | 167.2593 (314.7677, 28.0479) |
| Ghana | 380.4448 (688.3432, 68.9806) | 328.3997 (656.5016, 59.1020) | 2.4287 (4.9848, 0.4150) | 10765.8884 (18954.1701, 2037.6425) | 8816.8944 (17562.4739, 1691.7277) | 50.6026 (100.7715, 9.3173) |
| Guinea | 321.8338 (566.6526, 65.6477) | 579.5940 (1020.9053, 119.6456) | 11.4248 (20.2826, 2.3122) | 8068.0035 (14248.4922, 1669.0952) | 15166.1785 (26495.1350, 3284.7067) | 256.0625 (449.7824, 53.3341) |
| Guinea-Bissau | 81.4835 (138.1786, 16.6591) | 146.3077 (250.3118, 29.1869) | 22.8107 (39.3407, 4.2712) | 2359.6463 (4038.9547, 503.7173) | 4472.5703 (7664.5765, 933.9162) | 539.1303 (925.2423, 106.3599) |
| Liberia | 109.8178 (187.6291, 21.6235) | 264.8591 (480.0119, 58.9495) | 14.1667 (25.7998, 2.9707) | 2785.8581 (4784.7852, 572.0590) | 7579.0174 (13860.8420, 1794.3982) | 317.1010 (575.6611, 70.3128) |
| Mali | 212.2427 (373.1314, 41.5156) | 363.1911 (664.2812, 66.6377) | 4.8084 (8.8553, 0.8558) | 5871.2116 (10329.6125, 1159.8637) | 10040.9987 (18301.9678, 1914.1320) | 107.3501 (196.8711, 19.8546) |
| Mauritania | 124.1526 (221.8770, 23.2204) | 179.1637 (327.4208, 37.3656) | 9.4426 (17.4727, 1.9101) | 3156.5738 (5553.6030, 605.9050) | 4366.5249 (7870.0413, 960.0838) | 199.6963 (360.3774, 42.5533) |
| Niger | 226.6368 (409.3993, 45.3581) | 665.5873 (1186.4740, 150.4695) | 9.7389 (17.4163, 2.0950) | 6331.7126 (11229.8635, 1325.4245) | 17829.4540 (31196.2828, 4198.6622) | 212.4816 (375.9775, 48.4620) |
| Nigeria | 4448.7867 (8006.0634, 892.7976) | 6329.8986 (11105.4894, 1294.0817) | 8.3240 (14.7717, 1.6143) | 112606.0710 (199908.0956, 23297.6249) | 164032.5616 (284584.3207, 35646.2815) | 172.3629 (302.8746, 35.7259) |
| Sao Tome and Principe | 2.7494 (4.8537, 0.4987) | 4.2669 (8.2730, 0.8356) | 4.5302 (8.9316, 0.8738) | 63.8642 (111.8686, 11.7298) | 110.2544 (209.3308, 21.5919) | 94.3440 (181.4884, 18.5785) |
| Senegal | 153.4982 (287.5778, 27.8754) | 303.3709 (580.2968, 59.5339) | 4.6225 (8.7121, 0.8817) | 3940.8251 (7367.4220, 728.8824) | 7470.5953 (14011.5877, 1481.5567) | 95.3880 (181.7017, 19.0031) |
| Sierra Leone | 146.3768 (268.9085, 27.3323) | 230.2722 (433.4900, 41.6625) | 6.9441 (13.1495, 1.2363) | 3619.7769 (6561.7829, 708.9615) | 6135.7612 (11389.8099, 1135.2276) | 151.8309 (285.2885, 27.5914) |
| Togo | 127.5969 (218.7900, 24.6634) | 392.7414 (709.3399, 81.6367) | 12.1127 (21.6108, 2.4259) | 3546.0446 (6026.2660, 716.9377) | 11228.2202 (20269.5204, 2436.7183) | 271.2536 (490.0952, 56.2168) |
| American Samoa | 0.0726 (0.1897, 0.0103) | 0.4467 (1.0231, 0.0730) | 1.0993 (2.4966, 0.1773) | 2.1659 (5.3900, 0.2964) | 12.0217 (27.2469, 2.0721) | 25.2620 (58.1440, 4.3628) |
| Bermuda | 4.5346 (8.5431, 0.8301) | 1.7279 (3.6483, 0.2796) | 1.1308 (2.3533, 0.1812) | 96.2269 (180.5727, 18.8427) | 28.3117 (58.2189, 4.5445) | 21.0594 (43.0078, 3.4057) |
| Cook Islands | 0.2600 (0.5202, 0.0493) | 0.0126 (0.0444, 0.0008) | 0.0531 (0.1807, 0.0039) | 7.2600 (14.3923, 1.4314) | 0.2302 (0.7813, 0.0209) | 0.9992 (3.2963, 0.0950) |
| Greenland | 2.4540 (4.5735, 0.4702) | 1.3863 (2.7473, 0.2405) | 2.5319 (5.0061, 0.4321) | 70.1158 (127.0828, 14.5508) | 34.2703 (67.5204, 6.3689) | 51.8512 (101.6839, 9.2028) |
| Guam | 0.0325 (0.1142, 0.0012) | 0.0640 (0.2215, 0.0037) | 0.0297 (0.1026, 0.0020) | 0.7840 (2.6511, 0.0414) | 1.5365 (5.5406, 0.0981) | 0.7872 (2.7895, 0.0493) |
| Monaco | 1.6305 (3.3335, 0.2749) | 0.2705 (0.6472, 0.0377) | 0.2176 (0.5218, 0.0304) | 26.7077 (52.6533, 4.8202) | 3.7058 (8.7701, 0.5354) | 3.6527 (8.7098, 0.5590) |
| Nauru | 0.0235 (0.0641, 0.0031) | 0.1566 (0.3469, 0.0297) | 2.8950 (6.5884, 0.5321) | 0.8495 (2.3953, 0.1088) | 5.3637 (11.8265, 1.0815) | 75.1196 (166.5343, 14.4930) |
| Niue | 0.0587 (0.1183, 0.0100) | 0.0182 (0.0433, 0.0033) | 0.9291 (2.2646, 0.1700) | 1.2066 (2.5701, 0.2126) | 0.3881 (0.9256, 0.0754) | 19.5155 (45.3272, 3.8108) |
| Northern Mariana Islands | 0.0085 (0.0288, 0.0008) | 0.0790 (0.2262, 0.0118) | 0.2215 (0.5940, 0.0356) | 0.2978 (1.1070, 0.0259) | 2.0901 (5.9872, 0.3075) | 4.4145 (12.6141, 0.6819) |
| Palau | 0.0997 (0.2242, 0.0180) | 0.0961 (0.2496, 0.0143) | 0.6222 (1.5581, 0.0853) | 2.8637 (6.4083, 0.5104) | 2.5537 (6.5029, 0.3858) | 12.7745 (32.8242, 1.9932) |
| Puerto Rico | 240.5809 (446.5492, 44.3628) | 102.9419 (210.4606, 16.7406) | 1.2372 (2.4341, 0.2045) | 5095.9010 (9195.5591, 963.2605) | 1818.4063 (3553.6256, 298.3594) | 27.7695 (54.1758, 4.7274) |
| Saint Kitts and Nevis | 4.2055 (7.6525, 0.7836) | 1.3517 (2.6631, 0.2512) | 2.5221 (4.8689, 0.4449) | 90.5281 (159.8527, 17.0608) | 30.7285 (59.9232, 5.9884) | 47.5310 (92.9493, 9.1184) |
| San Marino | 1.4408 (2.7360, 0.2426) | 1.0208 (1.9899, 0.2057) | 1.0165 (1.9926, 0.2008) | 24.9994 (46.7896, 4.3614) | 15.7317 (30.3866, 3.0987) | 19.9705 (38.8691, 4.0240) |
| Tokelau | 0.0713 (0.1419, 0.0122) | 0.0319 (0.0676, 0.0055) | 2.2004 (4.6132, 0.3788) | 1.7768 (3.5401, 0.3058) | 0.7383 (1.5778, 0.1316) | 51.6332 (110.0489, 9.3691) |
| Tuvalu | 0.8172 (1.4899, 0.1674) | 0.6550 (1.3180, 0.1282) | 7.1365 (14.3202, 1.3887) | 25.2073 (46.3511, 5.3947) | 18.7111 (36.2013, 3.6303) | 177.8798 (341.9787, 34.7268) |
| United States Virgin Islands | 7.7786 (14.0991, 1.3819) | 4.3722 (8.9024, 0.6544) | 2.7226 (5.4507, 0.4190) | 200.4834 (357.7603, 38.0704) | 83.9085 (163.8319, 13.7289) | 54.6302 (103.7534, 9.0625) |
| South Sudan | 193.2551 (350.4475, 36.9487) | 394.7398 (728.7773, 83.4110) | 10.8893 (20.1829, 2.2076) | 5434.5984 (9837.4249, 1064.3471) | 12164.4253 (22142.2222, 2687.4985) | 269.7533 (496.7576, 57.0816) |
| Sudan | 4632.6198 (7766.9418, 988.3526) | 6480.8426 (11637.3252, 1396.5561) | 34.7952 (63.4155, 7.2453) | 137534.1896 (231469.7594, 30615.0980) | 191028.4285 (337333.7002, 42649.8782) | 835.9204 (1491.8553, 181.2205) |
